# Study on the Rapid Limit Test for Six Sulfonamide Residues in Food Based on the TLC-SERS Method

**DOI:** 10.3390/molecules29163977

**Published:** 2024-08-22

**Authors:** Yukun Ma, Min Zhang, Li Li, Jicheng Liu, Feng Xu, Yuanrui Wang, Bo Song, Tao Xu, Yue Hong, Honglian Zhang

**Affiliations:** 1 School of Pharmacy, Qiqihar Medical University, Qiqihar 161006, China; 17382849773@163.com (M.Z.); lilianlinsuo@163.com (L.L.); qyybliu@126.com (J.L.); 15845205504@qmu.edu.cn (F.X.); ihcpsu@qmu.edu.cn (B.S.); harvey-333@163.com (T.X.); lfy05222021@126.com (Y.H.); 2 Research Institute of Medicine and Pharmacy, Qiqihar Medical University, Qiqihar 161006, China; kuntengchongtian@163.com; 3 Qiqihar Institute for Food and Drug Control, Qiqihar 161006, China; wangyuanrui2021@163.com

**Keywords:** TLC-SERS, sulfonamides, residues, aquatic products, animal foods

## Abstract

Sulfonamides are not only widely applied in clinics but also highly valued in animal husbandry. Recently, it has become common for sulfonamide residues to exceed the standard limits in food, which can affect human health. Current regulations limit these residues. Therefore, we constructed a new limit test method to rapidly determine the levels of sulfonamide residues. Six sulfonamides were detected using the latest method called TLC-SERS, namely, sulfamethasone (A), sulfamethazine (B), sulfadoxine (C), sulfamethoxydiazine (D), sulfamethoxazole (E), and sulfathiazole (F). The optimal conditions for SERS detection were investigated for these six drugs, and the separation effects of different TLC spreaders on them were compared. Then, we successfully established a separation system using dichloromethane–methanol–ammonia in a ratio of 5:1:0.25 (*v*/*v*/*v*), which provided good separation effects on the six drugs. The residues were preliminarily separated via TLC. A silver sol solution was added to the spot on the silica gel G plate at the corresponding specific shift values, and SERS detection was performed. The sample solution was placed on the spot under a 532 nm laser, and the SERS spectrum was collected and analyzed for the six sulfonamides. The results showed obvious variations in the SERS spectrum among the six sulfonamides, with the LODs being 12.5, 6.4, 6.3, 7.1, 18.8, and 6.2 ng/mL from A to F, respectively, and an RSD of <3.0%. Within 48 h, the SERS signal for each sulfonamide drug was kept stable, with an RSD of <3.0%. The detection results of 20 samples using the TLC-SERS method were consistent with those obtained by UPLC-MS/MS. The established TLC-SERS method is simple and fast, providing a useful reference for the rapid detection of residue limits in food.

## 1. Introduction

Sulfonamides are representative synthetic antibacterial medicines that are not only widely used in clinical practice but also favored in animal husbandry [1,2,3]. Recently, it has become common for sulfonamide residues to be found in food, which may pose a serious threat to consumer health and carry potential risks of mutations and cancer. To eliminate counterfeit drugs and prevent excessive animal residues in food, it is necessary to develop simple and fast test methods.

The Chinese Pharmacopoeia (2020 edition) includes quality control methods for sulfonamide formulations, mainly using chemical methods such as HPLC, TLC, and UV-vis [4,5,6]. These methods are highly sensitive but have limited specificity, involve complex operations, and incur high experimental costs. Therefore, it is suggested that these methods would be difficult to implement for on-site drug identification experiments. To improve specificity, common foreign pharmacopeias, such as the United States Pharmacopoeia, use chromatography–infrared spectroscopy (IR) for the identification of formulations. However, excipients significantly interfere with the IR method, increasing the complexity and time required for sample pretreatment. In China, the national food standards mainly use the LC-MS method for determining sulfonamide residues in food, which offers high sensitivity and specificity [7]. However, sample pretreatment is cumbersome, the testing process is lengthy and costly, and it requires strict technical proficiency from operators, making it unsuitable for on-site rapid food detection. The Raman method, a recent analytical advancement, has high specificity and sensitivity [8,9], with almost no interference from moisture or silica gel. Based on Raman, surface-enhanced Raman scattering (SERS) has lower LODs, which could be applied to qualitative analysis and quantitative analysis for sulfonamide drugs in vitro or in vivo [10,11,12].

In the current study, a rapid identification method for six sulfonamide formulations was established using Raman spectroscopy. To further improve specificity and leverage the simple and rapid separation characteristics of TLC, an identification method for the active ingredients in common sulfonamide formulations was established using TLC–Raman combined technology. Finally, a limit detection method for the residues of six sulfonamides in food was established using TLC-conjugated SERS technology. These methods not only provide a new approach to improving current methods for medicine formulations but also provide a new reference basis for the rapid analysis of sulfonamide residues in food.

## 2. Results

### 2.1. Determination of the Characterization and Stability of Active Substrates

In this study, we obtained SERS substrates using the microwave method and performed the testing. Diluting the nanometer silver solution 14 times resulted in A = 0.742 at 424 nm (Figure S1a). It presented the results of electron microscopy detection (Figure S1b), which showed that the particles were ball-shaped. Additionally, the particle size distribution of the silver sol mainly ranged from 41.40 to 43.14 nm at different time points from 0 to 21 days (Figure S1c). The zeta potential ranged mainly from 31.14 to 35.80 mv (Figure S1d). Both the optical and morphological characteristics were conducive to the formation of hotspots and exhibited good stability.

### 2.2. Relative Rf and Raman

As shown in Table 1, there were notable differences in the Raman spectra of the six sulfonamide standard substances, with four characteristic peaks identified, namely, *ν*_CH2_, *β*_CH3_, *β*_C=C_, and *ν*_C-N_. Furthermore, the information from both the SERS and Raman spectra was compared to establish similarities, ensuring consistency between the two spectra. The first difference was the peak shape, and the second difference was the relative intensity of the characteristic peaks. Additionally, the total number of peaks in the SERS spectra was less than that in the Raman spectra. In summary, SERS detection effectively reflected the structural information of sulfonamides, enabling the simultaneous detection of all six sulfonamides.

### 2.3. In Situ SERS Detection of Sulfonamides

**Figure 1:**
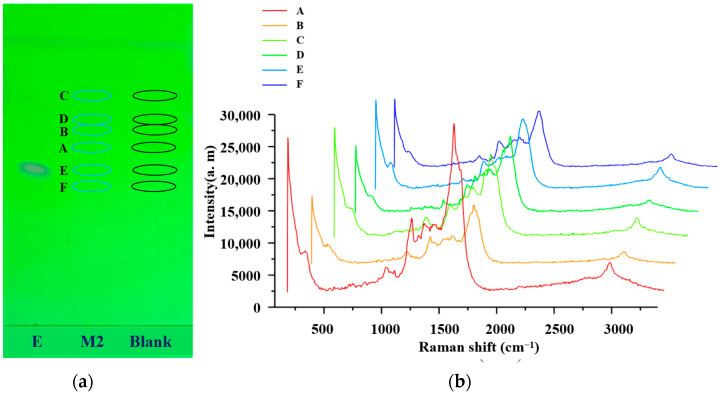
TLC and SERS charts of the 6 sulfonamides in mixed reference solution 2: (**a**) TLC chromatogram plots of the 6 sulfonamides in mixed reference solution 2; (**b**) SERS spectrum of the 6 sulfonamides in mixed reference solution 2.

There was an obvious spot in the TLC diagram of the reference solution of sulfamethoxazole, with Rf measured at 0.46. There were no spots in the TLC diagram of mixed reference solution 1. The positions of the other five sulfonamides were calculated and labeled according to their Rf and relative Rf values relative to sulfamethoxazole among the standard substances, as shown in Figure 1a. The relative Rf values for sulfamethasone (A), sulfamethazine (B), sulfadoxine (C), sulfamethoxydiazine (D), sulfamethoxazole (E), and sulfathiazole (F) were 1.20, 1.57, 1.73, 1.63, 1.00, and 0.83, respectively. Then, 6 μL of the silver sol solution was added to the marked positions, and the SERS spectra of the six sulfonamides are shown in Figure 1b. The same procedure was conducted at corresponding positions, and no SERS signal was observed at the blank position, indicating that the silver sol solution did not affect the SERS signal of the sulfonamides.

### 2.4. Calculation of EFs

An important index in this study was the Raman enhancement factor (EF), calculated using the formula EF = (I_SERS_/M_SERS_)/(I_blank_/M_blank_), where I_SERS_ and I_blank_ represent the Raman intensities at the characteristic peaks of the SERS-enhanced and blank substrates, and M_SERS_ and M_blank_ denote the mass (g) of sulfonamides on SERS-enhanced and blank substrates. The results are shown in Table 2.

### 2.5. Comparative Analysis of the Raman and SERS Results

As shown in Table 1, differences were observed in the Raman spectra of the six kinds of sulfonamides, with four characteristic peaks selected overall. Furthermore, the profiles of both the SERS and Raman spectra were generally consistent, with primary differences observed in peak shape and relative intensity. Additionally, the total number of peaks in the SERS spectra was less than that in the Raman spectra. In summary, SERS detection effectively reflects the structural information of sulfonamides, enabling the simultaneous detection of all six types.

### 2.6. Identification by SERS Combined with Relative Rf

**Figure 2:**
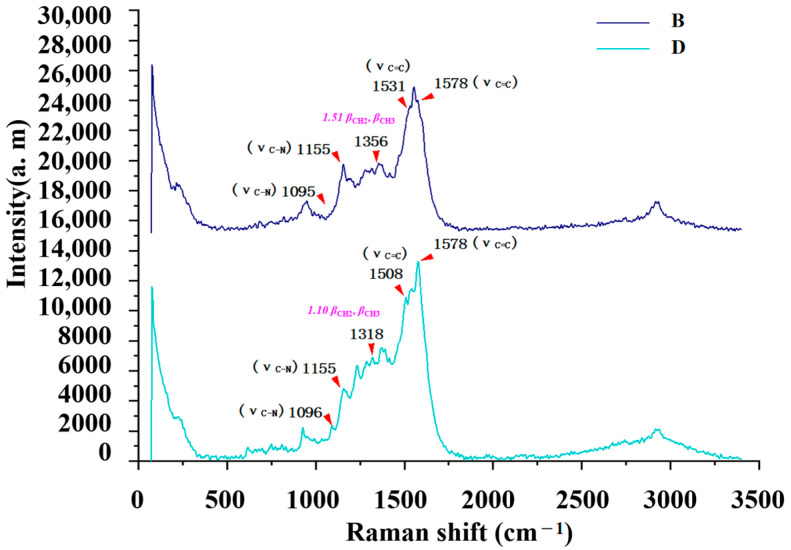
Comparative analysis diagram of sulfamethazine and sulfamethoxydiazine.

The TLC method was employed to separate six sulfonamides in this study, and the relative Rf was combined with SERS for sulfonamide analysis, offering higher sensitivity and specificity compared to the Raman method (Table 1). For example, the relative Rf values of sulfamethazine (B) and sulfamethoxydiazine (D) were 1.57 and 1.63, respectively, with a separation degree of 0.96. However, accurate localization using the TLC method alone was difficult. As shown in Figure 2, the relatively accurate localization of spots could be achieved based on the relative Rf values. When combined with SERS substrates, the signal intensity in the two spectra was amplified 1.4 × 10^4^ and 1.6 × 10^4^ times compared to that with Raman alone. For the same component, the relative peak intensity of the characteristic peaks was 1.51 and 1.10 for sulfamethazine (B) and sulfamethoxydiazine (D), respectively, further enhancing the specificity of the method.

### 2.7. LOD Test

**Figure 3:**
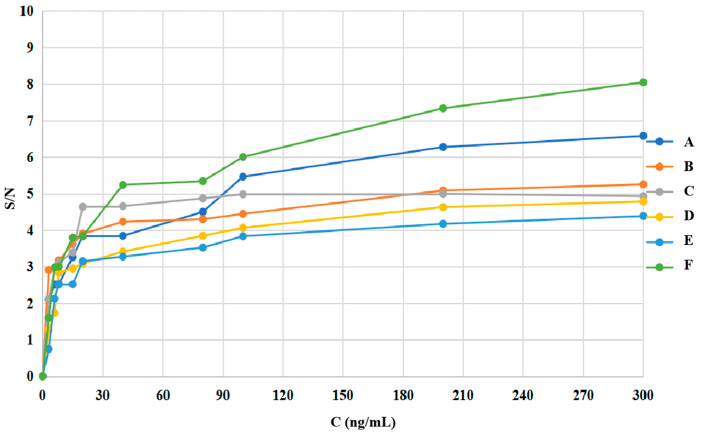
LOD curves of the TLC-SERS detection for the 6 kinds of sulfonamides.

The Raman signal was relatively stable because C-N was the unique functional group in the core structure of sulfonamides. Therefore, *ν*_C-N_ characteristic peaks were chosen for methodological validation. In Figure 3, the LODs of the six sulfonamides via SERS were shown based on the concentration (C) versus signal-to-noise ratio (S/N) curve, and the resulting values were listed in Table 3. We used S/N = 3 to predict the LODs.

According to the preparation method described in Section 3.5, the sulfonamide residues were extracted from food (2.0 g) using 500 μL of anhydrous ethanol. Considering the results in Table 3, the conversion formula between the MRL’ (μg/kg) and the specified standard MRL (ng/mL) was as follows: MRL = (MRL’ × 0.002 kg × 1000)/0.5 mL = 4 × MRL’.

Table 3 shows that the LOD was ≤MRL, indicating that the sensitivity of TLC-SERS meets the detection limit requirements for the six sulfonamides in food. We then evaluated whether the level of compounds exceeded the MRL.

### 2.8. Stability Assessment and Specificity Testing

TLC-SERS was used to measure the concentration of sulfonamide reference solutions at 0, 3, 6, 9, 12, 24, and 48 h. The concentrations were the same as the MRL, which was used to calculate the RSD% of peak heights for *ν*_C=C_, *β*_CH2_, *β*_CH3_, and *ν*_C-N_. As shown in Table 4, four characteristic peaks had an RSD ≤ 2.0% across the six sulfonamides, indicating the stability of the standard substances over 48 h. TLC-SERS was then used to sequentially measure 1000 ng/mL, 500.0 ng/mL, and the MRL of the sulfonamide reference solutions, which were measured three times. Table 5 shows the peak height at the characteristic peak (*ν*_C-N_) values. The results showed that the RSD of the peak height was ≤2.0% across the six sulfonamides, demonstrating the stability and suitability of the experimental instrument.

### 2.9. Simulated Positive Test

**Figure 4:**
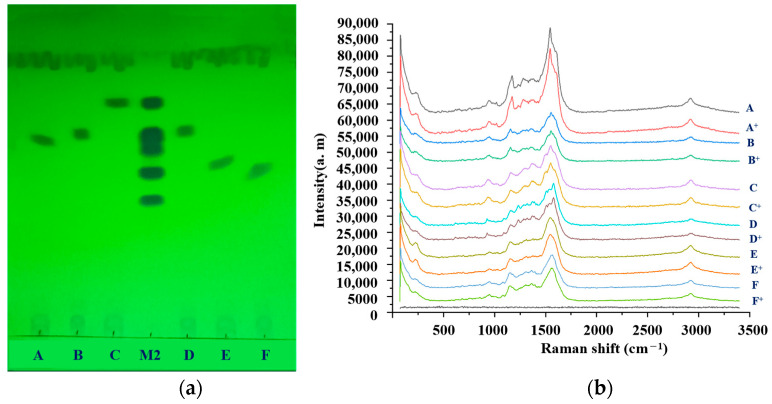
Specific results of the TLC-SERS detection for the 6 kinds of sulfonamides: (**a**) diagram of the TLC detection results; (**b**) SERS detection results.

All solutions, including mixed reference solution 1, as well as the simulated positive and negative sample solutions, were spotted in 10.0 µL quantities onto GF254 plates, as shown in Figure 4a. As shown in Figure 4b, the negative sample exhibited no signal, and no Raman signal was obtained in the SERS spectra of the simulated positive samples. The results confirmed that the matrix did not interfere with the limit experiments for detecting residues in the six sulfonamides, indicating that TLC-SERS offers high specificity and selectivity.

In Figure 4, A^+^ represented a simulated positive sample that contained sulfamethoxazole. B^+^ represented a simulated positive sample containing sulfamethoxazole. C^+^ was a simulated positive sample containing sulfadoxines. D^+^ represented a simulated positive sample, which would contain sulfamethoxazole. E^+^ represented a simulated positive sample, which would be added sulfamethoxazole. F^+^ represented a simulated positive sample that would contain sulfathiazole.

### 2.10. Limited Quantity Inspection Test of Real Samples

**Figure 5:**
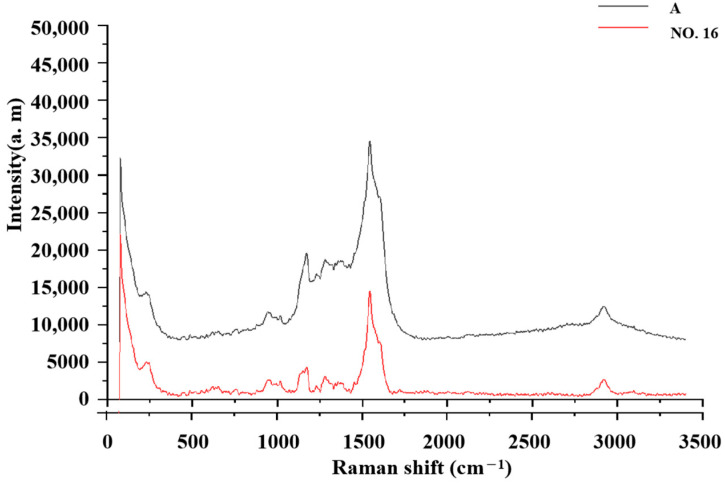
TLC-SERS detection results for the actual samples.

Based on the method described in Section 3.2, the results showed that most samples had no SERS signals; however, sample NO. 16 had the same SERS spectrum information as that in mixed reference solution 4. In Figure 5, it can be seen that the characteristic peaks of sample NO. 16 were lower than those of the control sample of sulfamethoxazole. The results indicated that the content of sulfamethoxazole in sample NO. 16 did not exceed the MRL (100 µg/kg), while the sulfonamides (sulfamethoxazole, sulfamethoxazole, sulfamethoxazole, and sulfathiazole) were not detected in other samples. According to China’s national food standards, the detection results of 6 sulfonamides were qualified among the 20 batches of samples by TLC-SERS.

### 2.11. Comparison of the Results of TLC-SERS and UPLC-MS Detection

To verify the accuracy of TLC-SERS, an authoritative analysis method (UPLC-MS) was used to quantitatively determine the residual content of sulfonamides in 20 batches of samples with possible residues. The results showed that the TLC-SERS detection limit method was accurate and reliable. The standard curves of five of the sulfonamides are shown in Table S1, with R^2^ values above 0.9990, indicating that the method was feasible and has a good linear relationship.

## 3. Materials and Methods

### 3.1. Materials 

All reagents were of analytical grade and were purchased from Merck KGaA, Darmstadt, Germany. The reference substances of sulfamethasone (100 mg/dose, 100,026–201,904, 99.7%), sulfamethoxazole (100 mg/dose, 100,411–201,902, 99.6%), sulfadoxine (50 mg/dose, 510,119–201,501, 99.2%), sulfamethoxydiazine (50 mg/dose, 510,095–201,401, 99.5%), sulfamethoxazole (50 mg/dose, 510,094–201,401, 99.6%), and sulfathiazole (50 mg/dose, 510,091–201,401 99.8%) were bought from the China Food and Drug Institute (labeled as A, B, C, D, E, and F, respectively), and anhydrous ethanol was used to dissolve sulfonamides. We used twenty batches of real samples of aquatic products from five different manufacturers (China), and the information is provided in Table S1. Anhydrous sodium sulfate was used to remove protein in the food, and acetonitrile was used to extract the sulfonamides in the food. Dichloromethane and methanol were used as developing agents in TLC. Silver nitrate and sodium citrate were used to prepare the active SERS substrates. TLC was performed using a thin-layer plate (Merck KGaA, Darmstadt, Germany) composed of high-performance silica gel and fluorescing additive F254. The plate is called a GF254 thin-layer plate and has a layer thickness of 0.2 + 0.03 mm, a particle size of 8 ± 2 um, and an aluminum carrier. The microinjector (10 μL) used for spotting on the thin-layer plates was purchased from Zhenhai Glass Instrument Factory, Ningbo, China.

### 3.2. Apparatus and Conditions

The equipment included DXRxi Raman spectrometer (Thermo Scientific, Waltham, MA, USA), Nicomp 380 ZLS nanoparticle analyzer (Shanghai Alfa Meijia Co., Ltd., Shanghai, China), HT 7700 transmission electron microscope (Beijing Shengjiachen Science and Trade Co., Ltd., Beijing, China), T6 UV visible spectrophotometer (Beijing Puxi General Instrument Co., Ltd., Beijing, China), SC-5 dynamic thin-layer chromatography instrument (Beijing Jinjian Zhiguang Pharmaceutical Confidence Technology Center, Beijing, China), Eppendorf Centrifuge 5417R desktop centrifuge (Thermo Scientific, USA), KQ-500 DB ultrasonic cleaner (Kunshan Ultrasonic Instrument Co., Ltd., Kunshan, China), QL-861 Vortex Instrument (Haimen Qilin Bell Instrument Manufacturing Co., Ltd., Haimen, China), PL-1000^+^ Micro Sampler (Mettler Toledo International Trade (Shanghai, China) Co., Ltd.), PL-200^+^ Micro Sampler (Mettler Toledo International Trade (Shanghai)) Limited company), HH-2 digital electronic constant temperature water bath (Guohua Electric Appliance Co., Ltd., Changzhou, China), AB135-S one hundred thousandth electronic balance (Mettler Toledo International Trade (Shanghai) Co., Ltd.), 204E one thousandth electronic balance (Mettler Toledo International Trade (Shanghai) Co., Ltd.), DHG-101-4A electric constant temperature blast drying oven (Gongyi Yuhua Instrument Co., Ltd., Gongyi, China), and GF_254_ thin-layer plate (Merck Group, Germany).

The laser wavelength was 532 nm; its intensity was 50%, and the laser power was 8 mw. The eyepiece magnification was 10 folds, and the number of scans was 20 times. The exposure time was 0.5 s. Using these settings, the Raman spectrum was recorded. Both OMNICxi Software (OMNIC 9.1.24, NXR FT Raman Software 9.1.0, OMNICMC 9.1.0) and Origin 2021 Software were used to process and analyze the spectra. SCIEX Qtrap 6500^+^ liquid chromatography–tandem triple quadrupole composite AB SCIEX linear ion trap mass spectrometry was used to verify the TLC-SERS limit test results of A~F in real samples. The chromatographic column was the Kromasil C_18_ column (100 × 2.1 mm × 1.8 µm). Mobile-phase A was a 0.1% formic acid aqueous solution, and mobile-phase B was a 0.1% formic acid acetonitrile solution. The elution process of the echelon was as follows: 0 min, 5% B, ~10 min, 95% B → 100%B, ~12 min, 100%B, 13 min, and 100%B → 5%B. The flow rate was 0.4 mL/min, the column temperature was 40 °C, and the injection volume was 1.0 μL. The electrospray ion source (ESI) was used to scan in multiple reaction monitoring (MRM) mode and detect in positive ion mode. The following settings were used: electric spray voltage: 5500 V, ion source temperature: 450.0 °C, curtain air (CUR): 20.0 psi, atomizing air (GS1): 45.0 psi; auxiliary gas (GS2): 45.0 psi; sulfamethasone (A), 251 → 155.9; sulfamethoxazole (B), 279 → 149; sulfadoxine (C), 311.2 → 156.1; sulfamethoxazole (D), 281.1 → 156.2; sulfamethoxazole (E), 268.2 → 113.1; and sulfathiazole (F), 256.3 → 107.9. The CE values of A~F were 21v, 19v, 25v, 24v, 20.5v, and 31v, respectively. The DP values of A~F were 15v, 35v, 30v, 27v, 50.5v, and 34.7v, respectively.

### 3.3. Preparation of Silver Nanoparticles

Silver nanoparticles were prepared to obtain the SERS values of the six sulfonamides, which were also called active SERS substrates. In this study, 56 mg of silver nitrate was precisely weighted at a certain ratio (2.7/1 = mL/mg), and 150 mL of water was added. Then, 4 mL of 1% sodium citrate solution was added to the solution. This study investigated the parameters of a silver sol, mainly including Amax, particle size, and potential.

### 3.4. Preparation of Reference and Mixed Reference Solutions

In the experiment, we prepared the sulfonamide A solution using the solution method, which required dissolving the reference substance (A) in anhydrous ethanol to a concentration of 1.000 mg/mL. The reference solutions of the other sulfonamides (B, C, D, E, and F) were prepared at a concentration of 1.00 mg/mL using the same method.

Mixed reference solution 1 was used for TLC determination by combining 1.000 mL of each of the reference solutions (A, B, C, D, E, and F) in the same container. Then, the mixture of the solutions was dried at a specific temperature (85 °C) and redissolved in 1.00 mL of anhydrous ethanol to obtain mixed reference solution 1.

Mixed reference substance solution 2 was prepared according to the MRL of sulfonamides provided in regulations by dissolving appropriate amounts of the six sulfonamides in the same amount of anhydrous ethanol. The final concentrations of sulfonamides A, B, C, D, E, and F were 400.0 ng/mL.

### 3.5. Preparation of Sample Solutions

A food sample of 2.00 g was precisely weighed. Both 10 g of Na_2_SO_4_ and 10 mL of anhydrous ethanol (1–100) were added to the same centrifuge tube. The residues were extracted from the food substrate via sonication for 15 min. After centrifugation at 4000 rpm and 4 °C for 5 min, the supernatant was passed through a 0.22 µm filter membrane to obtain the filtrate, which may contain the sulfonamides. The filtrate was concentrated in a water bath (85 °C) to approximately 1 mL and then transferred to a chromatography vial. All the solvent was evaporated in the water bath, and the sample solution was obtained by redissolving in 500.0 µL of anhydrous ethanol.

### 3.6. TLC Test

The TLC separation conditions for the six sulfonamides were as follows: Based on the thin-layer chromatography method (General Rule 0502) in 2020 ChP, 10.0 μL each of reference solutions 1-1 to 1-10 and mixed reference solution 1 were spotted onto the same plate. Then, dichloromethane–methanol–ammonia (5:1:0.25) was used as the developing agent, and the plate was removed and inspected under UV254. Sulfamethoxazole (E) was used as the reference to measure the relative Rf values of A, B, C, D, E, and F.

### 3.7. TLC-SERS

In the current study, TLC-SERS was used to test the levels of the six sulfonamide residues, offering a fast and highly specific method. During the TLC testing, 10.0 µL volumes of each of the standard solution and mixed standard solution 2 were added to the same plate. Then, fluorescence spots were observed under 254 nm conditions. In mixed reference solution 2, the concentration of the six sulfonamides was lower than the LOD value. However, only sulfamethoxazole had a very obvious spot in the TLC chromatogram with an Rf value of 0.46. The other five sulfonamides had no spots in the TLC plate of mixed reference solution 2. The positions of the other five sulfonamides could be determined based on the Rf and relative Rf values of the sulfonamide reference solution. Then, 6 μL of the silver sol solution was added to the marked areas, and the same procedure was conducted at the corresponding blank position. No SERS signal was clearly observed at the blank position, which suggests that the silver sol solution did not affect the SERS signal of the sulfonamides.

According to the above method, the sample solution was dropped onto the GF_254_ plate, and the residual substances were separated using TLC. The SERS values of the sulfonamides in the food could then be obtained. If the SERS spectrum of the ingredients in the food was consistent with the SERS of the corresponding reference substance in mixed reference solution 2, it indicated the presence of sulfonamide residues in the food. Based on these results, if the characteristic peaks were stronger than the corresponding control peaks, the levels of sulfonamide residues in the food exceeded the MRL, indicating unacceptable food quality. Conversely, if the characteristic peaks were not stronger, the sulfonamide residues in the food did not exceed related MRLs.

### 3.8. UPLC-MS/MS Validation

First, 1000.0 ng/mL of the mixed reference solution was sequentially diluted to concentrations of 750.0, 500.0, 450.0, 400.0, 300.0, 250.0, 200.0, 150.0, 100.0, 80.0, 50.0, 25.0, 12.5, and 6.25 ng/mL. After filtration through a 0.22 μm microporous filter membrane, the filtrate was transferred into brown sample vials. The mixed reference solutions were tested using the conditions outlined in Section 3.2, where a standard curve was generated with concentration on the *x*-axis and peak area on the *y*-axis. The test solution was prepared using the same method and analyzed under the same UPLC-MS/MS conditions. Finally, the concentration of sulfonamides in the test solution was determined by reading from the standard curve, thereby verifying the results obtained from TLC-SERS.

## 4. Conclusions

Based on Raman spectroscopy technology, this study focused on detecting residues of sulfonamides in food. We established the TLC-SERS method, which demonstrated the characteristics of high sensitivity, specificity, and selectivity, along with accuracy, reliability, and stability. Additionally, the method was simpler and faster than existing methods. The TLC-SERS experiment indicated a strong correlation between the SERS values of sulfonamides obtained through TLC-SERS. Distinction among the different types of sulfonamides was achieved based on the relative intensity and Raman shift of the characteristic peaks. A comparison of the results showed that the food matrix did not interfere with the detection of residues in the control solution, the simulated positive sample solution, and the negative sample solution.

The relative changes were measured according to the information from four characteristic peaks (*ν*_C=C_, *β*_CH2_, *β*_CH3_, *ν*_C-N_) of the same sample at various time points, with values of RSD ≤ 2.0%. Measuring different concentrations of sulfonamide standard substances revealed that the LOD of each sulfonamide was lower than or equal to the MRL. A comparison of the intensity between Raman and SERS for characteristic peaks showed that the EF for all sulfonamides was ≥1.1 × 10^4^. Additionally, the limited detection test confirmed no sulfonamide residues in the 20 patches of samples tested, with the results consistent with those from UPLC-MS/MS analysis. In summary, the proposed method offers a new approach for the rapid detection of harmful residue levels in food.

## Figures and Tables

**Table 1:** Comparative analysis of the Raman spectroscopy and SERS detection results for the six sulfonamides.

Formula/Relative R_f_	Raman Shift of the Blank Matrix (cm^−1^)/Relative Peak Intensity	SERS Shift (cm^−1^)/ Relative Intensity	Functional Group
A 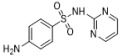 R_f_ = 1.20	3384~2999 (3 peaks) 1604~1104 (12 peaks) 1604/0.82 1510/0.15 1155/1.00 1104/0.34	3041~2802 (1 peaks) 1599~1018 (8 peaks) 1599/1.07 1544/1.54 1170/1.00 1091/0.31	Common peaks: *ν*_=CH_, *ν*_-CH2_, *ν*_-CH3_ Characteristic peaks: *ν*_C=C_ from phenyl rings *ν*_C=C_ from phenyl rings *ν*_C-N _ *ν*_C-N_
B 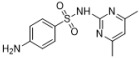 R_f_ = 1.57	3382~2858 (6 peaks) 1598~1096 (10 peaks) 1598/0.89 1513/0.12 1349/0.17 1149/1.00 1096/0.32	3035~2790 (1 peaks) 1578~1095 (7 peaks) 1578/2.80 1531/2.67 1356/1.51 1155/1.00 1095/0.30	Common peaks: *ν*_=CH_, *ν*_-CH2_, *ν*_-CH3 _ Characteristic peaks: *ν*_C=C_ from phenyl rings *ν*_C=C_ from phenyl rings *β*_CH2_, *β*_CH3 _ *ν*_C-N _ *ν*_C-N_
C 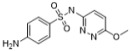 R_f_ = 1.73	3381~2956 (6 peaks) 1604~1100 (9 peaks) 1604/0.57 1514/0.15 1312/0.13 1153/1.00 1100/0.35	3078~2790 (1 peaks) 1591~1082 (7 peaks) 1591/1.72 1550/2.07 1365/1.18 1155/1.00 1082/0.24	Common peaks: *ν*_=CH_, *ν*_-CH2_, *ν*_-CH3 _ Characteristic peaks: *ν*_C=C_ from phenyl rings *ν*_C=C_ from phenyl rings *β*_CH2_, *β*_CH3 _ *ν*_C-N _ *ν*_C-N_
D 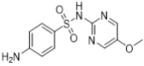 R_f_ = 1.63	3381~2946 (5 peaks) 1604~1100 (7 peaks) 1604/0.62 1515/0.18 1312/0.14 1154/1.00 1100/0.32	3064~2771 (1 peaks) 1592~1078 (6 peaks) 1578/2.45 1508/1.96 1318/1.10 1155/1.00 1096/0.24	Common peaks: *ν*_=CH_, *ν*_-CH2_, *ν*_-CH3 _ Characteristic peaks: *ν*_C=C_ from phenyl rings *ν*_C=C_ from phenyl rings *β*_CH2_, *β*_CH3 _ *ν*_C-N _ *ν*_C-N_
E 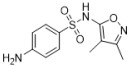 R_f_ = 1.00	3385~2938 (3 peaks) 1600~1097 (8 peaks) 1600/1.06 1502/0.41 1349/0.17 1158/1.00 1097/0.86	3055~2794 (1 peaks) 1576~1088 (6 peaks) 1573/2.31 1541/2.48 1338/1.52 1157/1.00 1088/0.39	Common peaks: *ν*_=CH_, *ν*_-CH2_, *ν*_-CH3 _ Characteristic peaks: *ν*_C=C_ from phenyl rings *ν*_C=C_ from phenyl rings *β*_CH2_, *β*_CH3 _ *ν*_C-N _ *ν*_C-N_
F 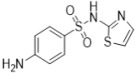 R_f_ = 0.83	3297~3060 (3 peaks) 1601~1097 (8 peaks) 1601/1.09 1536/0.39 1138/1.00 1097/0.31	3041~2812 (1 peaks) 1545~1080 (10 peaks) 1568/2.15 1545/2.02 1151/1.00 1080/0.19	Common peaks: *ν*_=CH_, *ν*_-CH2_, *ν*_-CH3 _ Characteristic peaks: *ν*_C=C_ from phenyl rings *ν*_C=C_ from phenyl rings *ν*_C-N _ *ν*_C-N_

Ν represents stretching vibration, and *β* represents in-plane bending vibration. The relative intensity of the peaks was obtained using the ratio of the absolute intensity to that of the reference peak, and the relative intensity of the reference peak was equal to 1.

**Table 2:** EFs of the SERS detection for the 6 kinds of sulfonamides.

Name	Functional Group	SERS				Raman				EF
		Raman Shift (cm^−1^)	I_SERS_	M_SERS_ (µg)	I_SERS_/M_SERS_	Raman Shift (cm^−1^)	I_blank_	M_blank_ (µg)	I_blank_/M_blank_	
A	*ν*_C=C_	1606	11,761	4.0 × 10^−3^	2.9 × 10^−6^	1602	1540	10	154.0	1.9 × 10^4^
	*ν*_C=C_	1523	9654	4.0 × 10^−3^	2.4 × 10^−6^	1513	465	10	46.5	5.2 × 10^4^
	*β*_CH2_, *β*_CH3_	1351	2506	4.0 × 10^−3^	6.3 × 10^−5^	1345	264	10	26.4	2.4 × 10^4^
	*ν*_C-N_	1158	4113	4.0 × 10^−3^	1.0 × 10^−6^	1154	213	10	21.3	4.8 × 10^4^
	*ν*_C-N_	1093	4200	4.0 × 10^−3^	1.1 × 10^−6^	1102	770	10	77.0	1.4 × 10^4^
B	*ν*_C=C_	1579	10,035	4.0 × 10^−3^	2.5 × 10^−6^	1598	716	10	71.6	3.5 × 10^4^
	*ν*_C=C_	1534	8516	4.0 × 10^−3^	2.1 × 10^−6^	1507	259	10	25.9	8.2 × 10^4^
	*β*_CH2_, *β*_CH3_	1376	2905	4.0 × 10^−3^	7.3 × 10^−5^	1349	524	10	52.4	1.4 × 10^4^
	*ν*_C-N_	1162	3111	4.0 × 10^−3^	7.8 × 10^−5^	1147	230	10	23.0	3.4 × 10^4^
	*ν*_C-N_	1088	905	4.0 × 10^−3^	2.3 × 10^−5^	1091	161	10	16.1	1.4 × 10^4^
C	*ν*_C=C_	1596	10,134	4.0 × 10^−3^	2.5 × 10^−6^	1602	314	10	31.4	8.1 × 10^4^
	*ν*_C=C_	1528	9365	4.0 × 10^−3^	2.3 × 10^−6^	1515	261	10	26.1	9.0 × 10^4^
	*β*_CH2_, *β*_CH3_	1372	3051	4.0 × 10^−3^	7.6 × 10^−5^	1357	519	10	51.9	1.5 × 10^4^
	*ν*_C-N_	1164	3731	4.0 × 10^−3^	9.3 × 10^−5^	1151	282	10	28.2	3.3 × 10^4^
	*ν*_C-N_	1089	4980	4.0 × 10^−3^	1.2 × 10^−6^	1098	288	10	28.8	4.3 × 10^4^
D	*ν*_C=C_	1575	10,014	4.0 × 10^−3^	2.5 × 10^−6^	1600	610	10	61.0	4.1 × 10^4^
	*ν*_C=C_	1532	8104	4.0 × 10^−3^	2.0 × 10^−6^	1506	220	10	22.0	9.2 × 10^4^
	*β*_CH2_, *β*_CH3_	1282	2412	4.0 × 10^−3^	6.0 × 10^−5^	1309	381	10	38.1	1.6 × 10^4^
	*ν*_C-N_	1168	3490	4.0 × 10^−3^	8.7 × 10^−5^	1158	217	10	21.7	4.0 × 10^4^
	*ν*_C-N_	1114	4510	4.0 × 10^−3^	1.1 × 10^−6^	1100	161	10	16.1	7.0 × 10^4^
E	*ν*_C=C_	1590	18,901	4.0 × 10^−3^	4.7 × 10^−6^	1592	523	10	52.3	9.0 × 10^4^
	*ν*_C=C_	1500	10,512	4.0 × 10^−3^	2.6 × 10^−6^	1498	83	10	8.3	3.2 × 10^5^
	*β*_CH2_, *β*_CH3_	1326	1320	4.0 × 10^−3^	3.3 × 10^−5^	1318	289	10	28.9	1.1 × 10^4^
	*ν*_C-N_	1158	8645	4.0 × 10^−3^	2.2 × 10^−6^	1164	128	10	12.8	1.7 × 10^5^
	*ν*_C-N_	1097	1693	4.0 × 10^−3^	4.2 × 10^−5^	1093	197	10	19.7	2.1 × 10^4^
F	*ν*_C=C_	1582	14,423	4.0 × 10^−3^	3.6 × 10^−6^	1588	367	10	36.7	9.8 × 10^4^
	*ν*_C=C_	1538	17,891	4.0 × 10^−3^	4.5 × 10^−6^	1540	188	10	18.8	2.4 × 10^5^
	*β*_CH2_, *β*_CH3_	1363	3597	4.0 × 10^−3^	9.0 × 10^−5^	1330	225	10	22.5	4.0 × 10^4^
	*ν*_C-N_	1151	7245	4.0 × 10^−3^	1.8 × 10^−6^	1149	105	10	10.5	1.7 × 10^5^
	*ν*_C-N_	1073	1728	4.0 × 10^−3^	4.3 × 10^−5^	1097	217	10	21.7	2.0 × 10^4^

**Table 3:** Comparison of the LODs and MRL for the 6 kinds of sulfonamide residues in food.

Compound	MRL’ (μg/kg)	LOD’ (μg/kg)	MRL (ng/mL)	LOD (ng/mL)
Sulfamethasone (A)	100.0	3.1	400.0	12.5
Sulfamethazine (B)	100.0	1.6	400.0	6.4
Sulfadoxine (C)	100.0	1.6	400.0	6.3
Sulfamethoxydiazine (D)	100.0	1.8	400.0	7.1
Sulfamethoxazole (E)	100.0	4.7	400.0	18.8
Sulfathiazole (F)	100.0	1.6	400.0	6.2

**Table 4:** Stability test results of the TLC-SERS detection for the 6 kinds of sulfonamides.

Compound	Characteristic Peak	Peak	Peak Intensity							RSD%
			0 h	3 h	6 h	9 h	12 h	24 h	48 h	
A	*ν*_C=C_	1589	12,378	12,409	12,433	12,298	12,192	12,385	12,180	0.8
	*ν*_C=C_	1541	20,387	20,386	20,451	20,299	20,261	20,189	20,377	0.4
	*ν*_C-N_	1170	13,238	13,301	13,221	13,298	13,212	13,245	13,210	0.3
	*ν*_C-N_	1094	4101	4067	3950	4043	4131	4043	4013	1.5
B	*ν*_C=C_	1579	30,482	30,501	30,512	30,497	30,498	30,677	30,479	0.2
	*ν*_C=C_	1527	29,067	29,087	29,101	29,087	29,076	29,054	29,081	0.1
	*β*_CH2_, *β*_CH3_	1353	16,438	16,442	16,428	16,451	16,387	16,459	16,432	0.1
	*ν*_C-N_	1151	10,886	10,874	10,890	10,881	10,876	10,873	10,898	0.1
	*ν*_C-N_	1096	3266	3247	3259	3275	3269	3184	3190	1.2
C	*ν*_C=C_	1593	26,710	26,687	26,736	26,692	26,714	26,721	26,698	0.1
	*ν*_C=C_	1551	32,145	32,157	32,210	32,152	32,163	32,171	32,156	0.1
	*β*_CH2_, *β*_CH3_	1367	18,324	18,318	18,319	18,325	18,301	18,327	18,330	0.1
	*ν*_C-N_	1160	15,529	15,527	15,531	15,537	15,547	15,534	15,512	0.1
	*ν*_C-N_	1082	3727	3736	3743	3729	3736	3721	3719	0.2
D	*ν*_C=C_	1573	34,198	34,210	34,201	34,199	34,209	34,211	34,312	0.1
	*ν*_C=C_	1510	27,356	27,362	27,340	27,367	27,382	27,374	27,396	0.1
	*β*_CH2_, *β*_CH3_	1316	15,354	15,462	15,341	15,367	15,344	15,361	15,349	0.3
	*ν*_C-N_	1154	13,958	13,966	13,973	13,881	13,941	13,970	14,163	0.6
	*ν*_C-N_	1097	3350	3353	3367	3341	3226	3359	3461	2.0
E	*ν*_C=C_	1573	18,711	18,701	18,698	18,687	18,723	18,691	18,707	0.1
	*ν*_C=C_	1542	20,088	20,095	20,067	20,087	20,075	20,074	20,064	0.1
	*β*_CH2_, *β*_CH3_	1339	12,312	12,269	12,328	12,300	12,268	12,276	12,293	0.2
	*ν*_C-N_	1161	8120	7969	8128	7900	8168	8176	7993	1.4
	*ν*_C-N_	1087	3160	3169	3128	3170	3168	3176	3189	0.6
F	*ν*_C=C_	1560	41,031	40,976	40,986	40,989	41,026	41,028	41,020	0.1
	*ν*_C=C_	1544	38,549	38,551	38,539	38,543	38,611	38,563	38,557	0.1
	*ν*_C-N_	1149	19,084	19,089	19,094	19,076	19,079	19,187	19,093	0.2
	*ν*_C-N_	1076	3626	3639	3617	3620	3649	3623	3645	0.4

**Table 5:** Repeatability test results of the TLC-SERS detection for the 6 kinds of sulfonamides.

Compound	Characteristic Peak	Peak	Intensity 1	Intensity 2	Intensity 3	RSD%
A	*ν*_C-N_	1094	7561	7503	7693	1.3
	*ν*_C-N_	1096	4709	4740	4801	1.0
	*ν*_C-N_	1094	4104	4177	4113	1.0
B	*ν*_C-N_	1098	6521	6504	6374	1.2
	*ν*_C-N_	1100	4636	4667	4604	0.7
	*ν*_C-N_	1096	3266	3191	3154	1.8
C	*ν*_C-N_	1178	4073	3985	3957	1.5
	*ν*_C-N_	1180	3849	3770	3770	1.2
	*ν*_C-N_	1182	3727	3702	3562	2.4
D	*ν*_C-N_	1096	10,158	10,368	10,220	1.1
	*ν*_C-N_	1098	4814	4741	4744	0.9
	*ν*_C-N_	1097	3350	3242	3243	1.9
E	*ν*_C-N_	1084	8005	8165	7840	2.0
	*ν*_C-N_	1092	5973	5758	5882	1.8
	*ν*_C-N_	1087	3159	3206	3149	1.0
F	*ν*_C-N_	1081	8073	7830	7951	1.5
	*ν*_C-N_	1079	5036	5237	5183	2.0
	*ν*_C-N_	1076	3626	3611	3687	1.1

## Data Availability

The original contributions presented in the study are included in the article, further inquiries can be directed to the corresponding authors.

## Supplementary Materials

The following supporting information can be downloaded at: https://www.mdpi.com/article/10.3390/molecules29163977/s1, Figure S1: Characterization results of the silver sol solution. (a) UV absorption spectrum of the silver sol solution; (b) appearance of the silver sol solution; (c,d) particle size of the silver sol solution; (e) zeta potential of the silver sol solution. Table S1: Information for 20 batches of samples in the current study.
