# Supplementary material for: Study on the Rapid Limit Test for Six Sulfonamide Residues in Food Based on the TLC-SERS Method
Source: Molecules. 2024 Aug 22;29(16):3977. doi: 10.3390/molecules29163977 (PMC11357332; doi:10.3390/molecules29163977)
Supplement: Supplementary file 1 [file molecules-29-03977-s001.zip › molecules-3064286-supplementary.pdf]

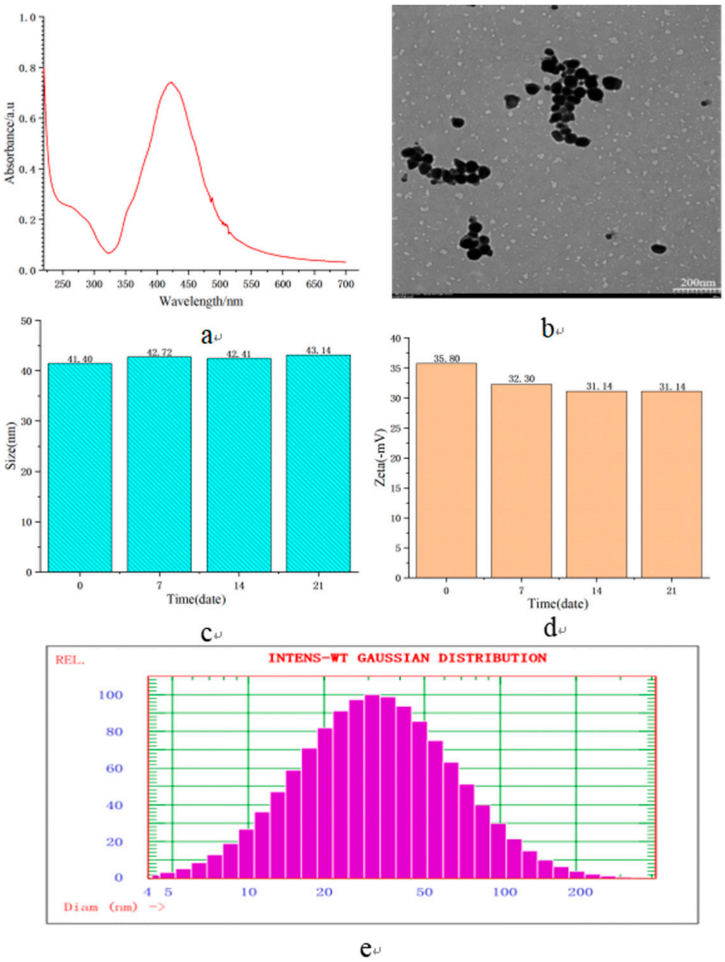

Figure S1 Characterization results of silver sol solution

a. UV absorption spectrum of silver sol solution b. appearance of silver sol solution

c-d. particle size of silver sol solution e. Zeta potential of silver sol solution

Table S1 Information for 20 batches of samples in the current study

NO.	Sample Name	NO.	Sample Name
S1	Chicken	S11	Squid
S2	Goose meat	S12	Carrying fish
S3	Beef	S13	Silver carp
S4	Pig liver	S14	Mirror fish
S5	Mutton	S15	Crucian
S6	Heart-shaped	S16	Sea bream
S7	Pork	S17	Salmon
S8	Egg	S18	Crystal fish
S9	Herring	S19	Yellow croaker
S10	Cod fish	S20	Shrimp
